# Fully Biobased Epoxy Resins from Fatty Acids and Lignin

**DOI:** 10.3390/molecules25051158

**Published:** 2020-03-05

**Authors:** Pablo Ortiz, Richard Vendamme, Walter Eevers

**Affiliations:** 1 Flemish Institute for Technological Research–VITO, Separation & Conversion Technology, Boeretang 200, 2400 Mol, Belgium; richard.vendamme@vito.be (R.V.); walter.eevers@vito.be (W.E.); 2 Biorizon, Auvergnedijk 2, 4612 PZ Bergen op Zoom, The Netherlands; 3 Department of Chemistry, University of Antwerp, Groenenborgerlaan 171, B-2020 Antwerpen, Belgium

**Keywords:** lignin, lignocellulose, aromatics, biobased, epoxy, fatty acid, biopolymers, biobased materials, biorenewable

## Abstract

The use of renewable resources for plastic production is an imperious need for the reduction of the carbon footprint and the transition towards a circular economy. With that goal in mind, fully biobased epoxy resins have been designed and prepared by combining epoxidized linseed oil, lignin, and a biobased diamine derived from fatty acid dimers. The aromatic structures in lignin provide hardness and strength to an otherwise flexible and breakable epoxy resin. The curing of the system was investigated by infrared spectroscopy and differential scanning calorimetry (DSC). The influence of the different components on the thermo-mechanical properties of the epoxy resins was analyzed by DSC, thermal gravimetric analysis (TGA), and tensile tests. As the content of lignin in the resin increases, so does the glass transition, the Young’s modulus, and the onset of thermal degradation. This correlation is non-linear, and the higher the percentage of lignin, the more pronounced the effect. All the components of the epoxy resin being commodity chemicals, the present system provides a realistic opportunity for the preparation of fully biorenewable resins at an industrial scale.

## 1. Introduction

We live surrounded by plastics: from packaging to clothing, including construction materials, components of vehicles, furniture, electronics, and an endless list of commodities. In these days of banishment of plastics, their benefits should be kept in mind. For example, the use of plastics for automotive parts results in lighter vehicles that consume less fuel, therefore reducing the greenhouse emissions [1]. However, the overall carbon footprint of most plastics is high because their production relies on fossil fuels. The use of renewable resources for plastic production is, therefore, an imperious need. It is a challenging endeavor because the high performance of current fossil-derived plastics has set a high bar for biobased plastics. The replacement of petrol-derived monomers by their biobased analogs offers, in principle, a win-win situation; that is, the same characteristics and processing as fossil-derived plastics but derived from renewable sources. However, direct replacement is commonly not the most (resource and waste) efficient option because the biomass-derived building blocks are different from the petrol-derived ones [2]. Furthermore, although possible in some cases (as in the case of polyethylene), few of the current industrially produced biobased polymers have a 100% biomass content [3]. Currently, the use of new, biomass-derived building blocks for the production of fully biobased polymers is practically restricted to academia. Unfortunately, the price and/or availability of the monomers employed in these studies are a great obstacle for their industrial application [4].

**Scheme 1:**
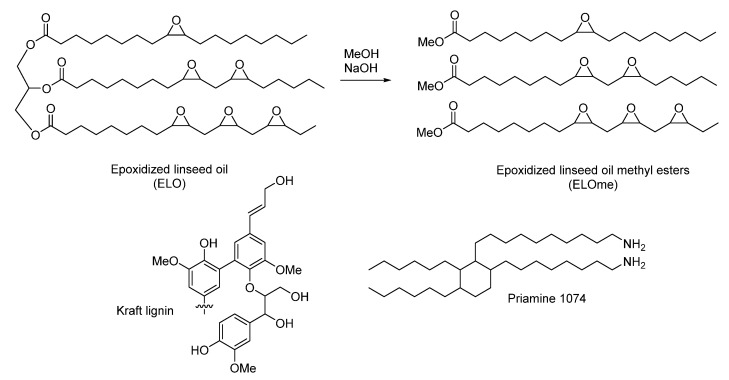
Biobased building blocks used in this study to produce biobased epoxy resins.

Here, we report a fully biobased epoxy resin from biorenewable bulk chemicals: lignin and vegetable oils [5]. Currently, the glycidyl ether of bisphenol A is the main epoxy monomer, and it is synthesized from BPA and epichlorohydrin. There have been many attempts to replace BPA with biobased aromatic structures and subsequently cure it [6]. This approach, however, incurs the above-mentioned limitations, namely not being fully biobased (usually due to the curing agent) and the exotic monomers that are used [7]. Some of the few biobased epoxy building blocks that reach industrial-scale production are two epoxidized vegetable oils (EVO), namely epoxidized soybean oil (ESO) and epoxidized linseed oil (ELO) (Scheme 1). The possibility of using epoxidized vegetable oils (EVO) in epoxy polymers has been known for decades, but due to the lack of rigid structures (cycloaliphatic, aromatic) the resulting polymers had a low strength [8].

Lignin, with its highly cross-linked phenolic network (Scheme 1), could act as a counterbalance for that deficit of stiffness. Moreover, lignin is currently a waste product from the pulp and paper industry and the only biorenewable source of aromatics that can provide enough feedstock to replace petrol-based aromatics. Technical (industrially produced) lignins are currently the only lignins available at a large scale. Among them, the Kraft process is the dominant in the pulp and paper industry (Scheme 1) [9]. By the use of high temperatures and basic conditions, lignin and hemicellulose are separated from cellulose. High-quality cellulose is obtained, but the resulting lignin is altered. In the pulp and paper industry, this is not a problem because lignin is burned to generate energy and to recover inorganic chemicals. However, the use of this lignin for polymer synthesis represents a challenge due to high polydispersity, limited solubility, and heterogenicity [10]. The fractionation of kraft lignin (KL) [11,12] is an interesting technique that has successfully been applied for epoxy resins synthesis [13]. In epoxy resins, the catalog of hardeners that can be used to cure epoxy resins is broad, the polyamines being the most common choice. Unfortunately, the industrial production of biobased polyamines is still in its infancy [7,14]. One of the few marketed biobased diamines is Priamine^®^ from Croda, derived from dimerized fatty acids (Scheme 1).

## 2. Results and Discussion

### 2.1. Design of a Fully Biobased Epoxy Resin

The solubility tests of Lignoboost^®^ KL showed that it was fully soluble in tetrahydrofuran, partially soluble in methanol and acetone but not quite in ethyl acetate. Consequently, methanol and acetone were chosen as fractionation solvents, and the properties of the extracted lignin were analyzed (Table 1 and Supplementary Materials S1–S6).

The methanol extracted fraction, KL_MeOH_, with the lowest polydispersity index (PDI), molecular weight (Mw), and glass transition (T_g_), was chosen for the biopolymer development. Initial efforts were directed towards using lignin as a curing agent for the epoxy group. The quest for a lignin-epoxy resin goes decades back [15,16]. There is an extensive body of literature showing that the hydroxyl groups in the lignin can react with terminal epoxy groups in molecules such as (polyethelene) glycerol digylicidy ether [17,18,19,20,21]. However, EVO such as ESO or ELO did not yield a fully cured product, probably due to the lower reactivity of internal epoxy groups. Consequently, the addition of a second curing agent, namely a biobased diamine derived from dimer fatty acids (Priamine 1074 ^®^) was considered and successfully tested. The miscibility of the non-polar oils with polar lignin was not always optimal but could be addressed by transesterifying the EVO with methanol (Scheme 1). Apart from improving the compatibility of the mixture, the use of methyl esters of EVO (ESO_me_ and ELO_me_) led to samples with higher T_g_ than those prepared with the EVO. Although the transesterification process was carried out in the lab, it is worth noting that there are commercial suppliers of ESO_me_ and ELO_me_.

### 2.2. Possible Curing Mechanism

**Figure 1:**
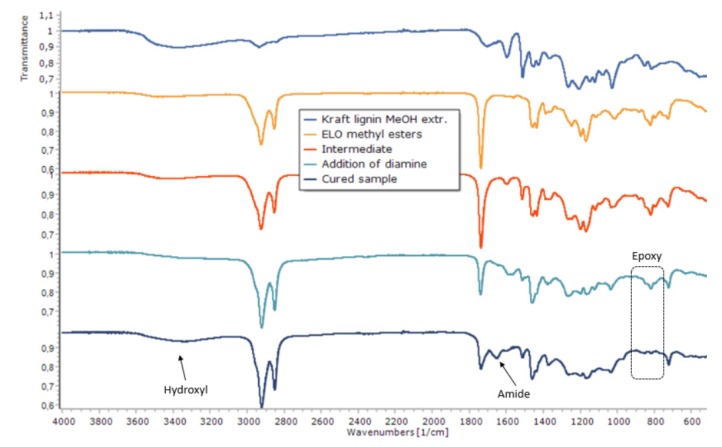
Infrared spectra of the starting materials and the evolution of the mixture during the curing process.

**Scheme 2:**
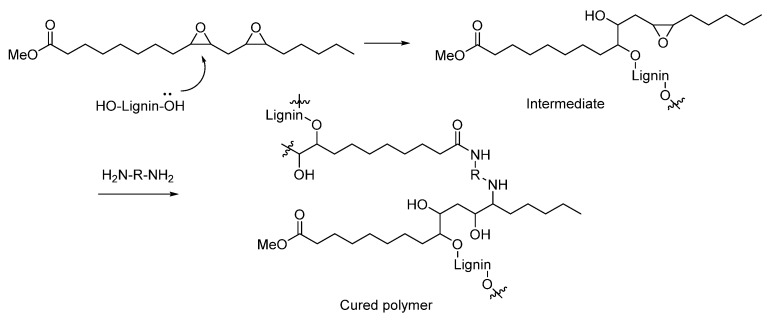
Proposed mechanism of the curing of the epoxy resins.

**Figure 2:**
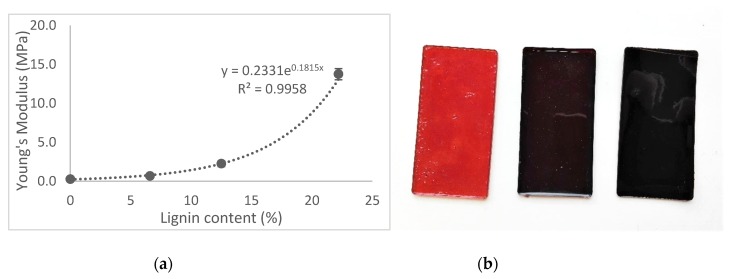
(**a**) Correlation between the lignin content and Young’s modulus of the cured epoxy resins. (**b**) Physical appearance of epoxy resins with 0%, 12.5%, and 22.2% weight of KL_MeOH_.

The reactivity of epoxy groups towards hydroxyl and amine groups is well understood [22], and its curing can be followed using infrared spectroscopy [13]. In our system, besides the epoxy and the amine groups, the hydroxyl groups from lignin were also present. In order to better understand the interaction between the three components, the curing mechanism was studied. More specifically, we wanted to prove that the lignin reacts with the EVO and that it gets incorporated in the structure. Our hypothesis was that the reaction of lignin with an excess of EVO led to an intermediate with epoxy groups still available to be cured upon addition of the diamine (Figure 1 and Scheme 2) [19]. In order to prove that the lignin reacted with the epoxide groups and was not merely embedded in the cured epoxy matrix, the curing of the resin was analyzed by DSC and an exotherm was observed (Supplementary Materials S7). This is in line with previous research [20]. Furthermore, a cured sample was immersed in tetrahydrofuran and kept for 48 h. Afterwards, the epoxy resin was removed, and the liquid was analyzed by ^1^H NMR. It showed that the leaching corresponded to the diamine and not to lignin (Supplementary Materials S8). This result is not surprising considering that an excess of diamine in relation to epoxides was used. IR showed the disappearance of the stretch corresponding to the epoxy group at 825 cm^−1^ as well as the increase of the broad hydroxyl stretch between 3200 and 3500 cm^−^^1^ (Figure 2). To our surprise, we noticed that the curing with diamine resulted in the appearance of another peak at around 1650 cm^−1^ (Figure 1). We identified it as that of the C=O stretch of the amide, resulting from the reaction of the ester of EVO with the excess of diamine (Scheme 2).

### 2.3. Influence of the Dimer Diamine Content

This finding encouraged us to further explore the effect of the amount of the diamine on the properties of the epoxy resins. Fixing the amount of lignin to half the weight of the ELO_me_, various equivalents of diamine to epoxy equivalents in ELO_me_ were tested (Table 2). The Young’s modulus (E_Young_) was exponentially correlated (Supplementary Materials S9) with the amount of diamine, which is understandable considering its long alkyl chain. Interestingly, other thermomechanical properties did not follow the same trend. Both the tensile strength and glass transition temperature gave similar results for all the samples but one. When one equivalent of diamine was used, the sample had a higher strength and T_g_. These results suggest that with one equivalent of diamine, the cross-linking is the highest. Furthermore, they reinforce the proposed curing mechanism. Because some of the OH groups of lignin reacted with the epoxy groups of the EVO, the one equivalent of the diamine was actually in excess to the epoxy groups of the EVO, and the higher cross-linking must have come from the amide formation.

### 2.4. Influence of the Lignin Content on the Mechanical Properties

Consequently, we fixed the amount of diamine to one equivalent to the epoxy groups in the ELO_me_, and we moved on to explore the influence of the KL_MeOH_ content on the thermomechanical properties of the resulting polymers (Table 3). Both Young’s modulus and the tensile strength showed an exponential increase with an increasing amount of lignin (Figure 2a). Because of the long aliphatic chains of both ELO_me_ and the diamine, the polymer resulting from their mixture was an elastic although easily breakable material. The incorporation of lignin dramatically changed the properties, and with 12.5% weight of KL_MeOH_ in the composition, the Young’s modulus increased ten-fold. Notably, doubling that amount of lignin resulted in a fifty-fold increase in E_Young_ compared with the sample without lignin. Testing the limits, a resin with an even higher percentage of lignin (36.4%) was also tested but resulted in a brittle sample. Besides the mechanical properties, the effect of lignin was also noticeable in the color of the samples, which ranged from dark orange to dark brown (Figure 2b).

### 2.5. Influence of the Lignin Content on the Thermal Properties

**Figure 3:**
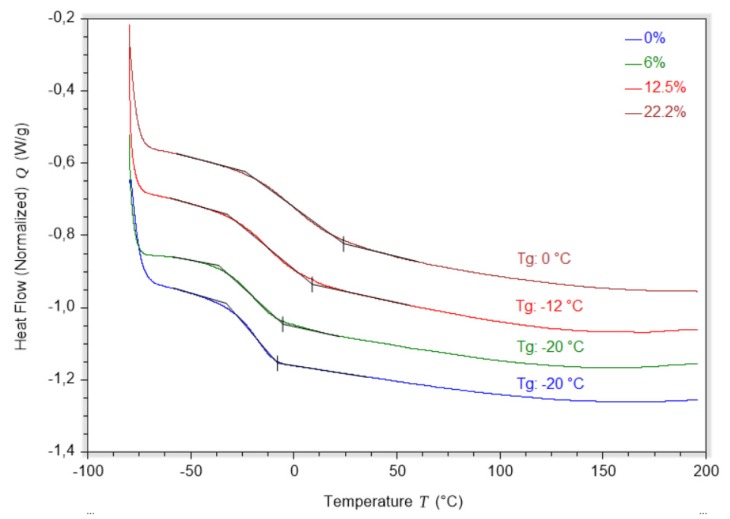
Influence of the KL_MeOH_ content (expressed as weight%) on the T_g._

Lignin also had a marked effect on the T_g_. The cross-linked epoxy resin resulting from the curing between the ELO_me_ and the diamine showed a glass transition temperature of −20 °C (Figure 3). This result is in line with previous research on epoxy resins derived from EVO and diamines derived from fatty acids [23]. As with the mechanical properties, the effect of KL_MeOH_ on the glass transition followed an exponential increase: 6% of lignin had no influence, but 12.5% of KL_MeOH_ resulted in a T_g_ of −12 °C, and the resin with 22.2% of lignin had a glass transition of 0 °C (Figure 3). A similar exponential trend had been observed before for an epoxy-lignin-amine system [19]. In that case, the epoxy component was polyethelene glycerol digylicidy ether and the amine triethylene tetramine. Despite the differences with our system, the glass transitions are surprisingly close for a defined amount of lignin. This fact underlines the strong impact lignin has on the thermal properties of epoxy resins.

**Figure 4:**
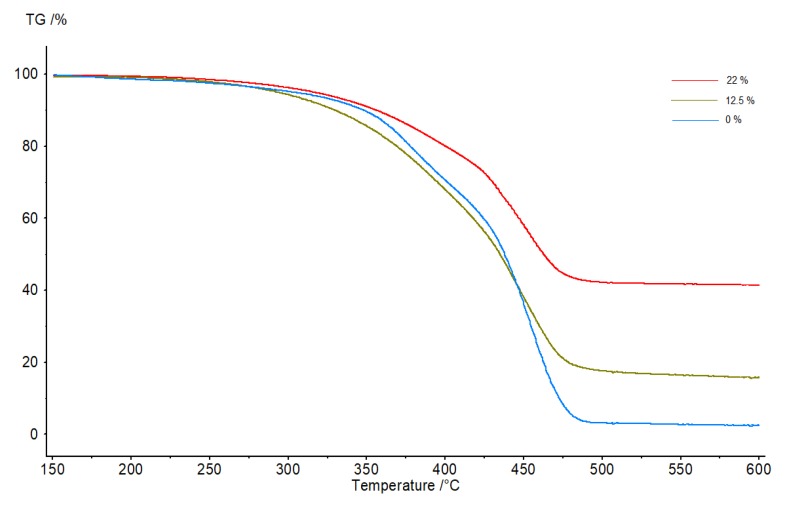
The TGA curves of epoxy resins with an increasing amount of KL_MeOH_ (expressed as weight %).

Likewise, the presence of KL_MeOH_ had a profound impact on the thermal stability of the resins. Although there are some exceptions [24], the incorporation of lignin in a polymeric matrix typically leads to increased thermal stability [25]. TGA experiments (Figure 4 and Table 4) showed increased stability as the content of lignin in the sample rose. The sample without lignin was completely consumed at 500 °C, which is in agreement with previous research on EVO/dimer systems [23]. By contrast, the sample with 12.5% of KL_MeOH_ in weight showed 14% of residual weight. Importantly, the residual weight cannot just be attributed to the remaining lignin, as the sample with 22% weight of KL_MeOH_ retained 40% of weight at 500 °C. Interestingly, the same exponential effect to that of the Young modulus of the resins was also observed here. That is, the effect from doubling the lignin content (from 12.5% to 22% of the total weight) had a greater impact than that from not having lignin at all to having a 12.5% content.

### 2.6. Influence of the Type of Lignin on the Thermomechanical Properties

After the observed strong effect of the amount of lignin on the thermomechanical properties of the resins, we wondered how these would be influenced by the characteristics of lignin (Table 5). Different fractions of KL showed a positive correlation between the Mw and the Young’s modulus, in line with previous reports [13]. That is, the higher the Mw, the higher the Young’s modulus. However, no such trend was observed for the ultimate tensile strength (Σ_Break_) or elongation at break (Ε_Break_). The T_g_ of the epoxy resins made from different Kraft lignins had a remarkably similar value, and only that of the acetone extracted fraction was slightly higher than the rest. A similar effect had been noted before for lignin-based epoxy resins [13].

One of the features of lignin is the variability and heterogenicity among different samples, and therefore we were interested in comparing a KL from another source. West Fraser KL led to epoxy resins with not striking but noticeable differences. The resin containing West Fraser KL had a slightly lower Young’s modulus than Lignoboost KL, which points again to a relation between the Mw and the Young’s modulus. However, this seems to not be a linear relation, because West Fraser KL had a Mw of 4080 but a E_Young_ lower than KL_Acetone_, which had a Mw of 2540. Despite its relatively low Young’s modulus, the epoxy resin made from West Fraser KL showed the highest Σ_Break_ and Ε_Break_ of the series.

**Figure 5:**
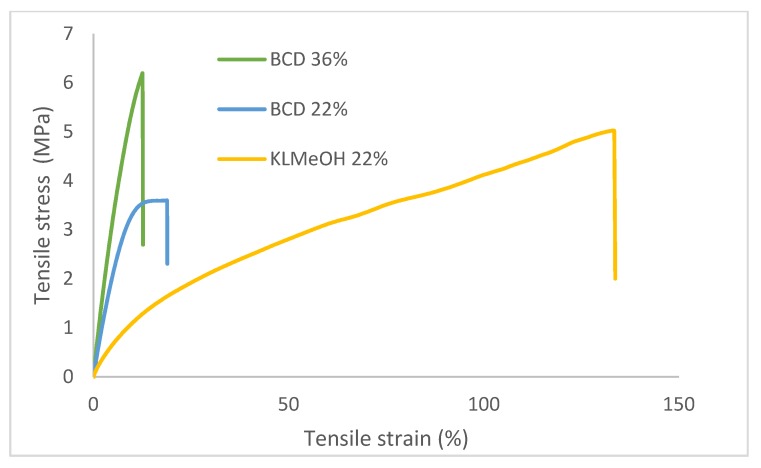
Selected tensile stress-strain curves of different cured epoxy resins.

Fractionation of technical lignins is a good approach to reduce polydispersity and Mw, thus allowing for better miscibility and reactivity in the prepolymer mixture. Even lower molecular weight fractions can be obtained from the depolymerization of lignin. These streams are attractive because they can be in oily form and thus directly blended with the other components without the need for solvents [10]. To that end, solvent-extracted, base-catalyzed depolymerization (BCD) oil from Eucalyptus, with a Mw of 330 and PDI of 1.4, was tested. The polymer resulting from the incorporation of 22% of such lignin showed a T_g_ of 9 °C, significantly higher than the samples prepared with KL. Moreover, the structural differences of lignin had a great impact on the mechanical properties, leading to harder epoxy resins (higher Young’s modulus and lower Ε_Break_, Table 5 and Figure 5). Contrary to the case of KL, where reducing the Mw and polydispersity led to samples with a lower E_Young_, the low Mw of BCD lignin oil did not result in a less stiff material. Moreover, the use of such lignin oil allowed us to increase the content of lignin. A sample with 36.4% of lignin content could be prepared, which displayed a Young’s modulus of 60 MPa and a T_g_ of 15 °C.

## 3. Materials and Methods

### 3.1. Chemicals

Epoxidized soybean oil (Efka^®^ PL 5382) was kindly provided by BASF; Epoxidized linseed oil (Lankroflex™) was kindly provided by Valtris. Dimer diamine (Priamine 1074^®^) was kindly provided by Croda (AHEW 138g/equiv.). Lignoboost^®^ Kraft lignin was provided by Innventia Institute. West Fraser Kraft lignin was kindly provided by West Fraser. Base-catalyzed depolymerized (BCD) solvent-extracted eucalyptus lignin oil was kindly provided by Fraunhofer Institute. Tetrahydrofuran was purchased from Fisher Scientific, methanol and ethanol from Acros Organics, and 1-methylimidazol from Alfa Aesar. Sodium hydroxide was purchased from Sigma-Aldrich.

### 3.2. Transesterification of EVO

The formation of methyl esters of epoxidized soybean and linseed oil was carried out following a modified literature procedure [26]: 2 g of NaOH were dissolved in 50 mL of methanol and transferred to a round bottom flask containing 100 g of epoxidized vegetable oil. 5 mL of dichloromethane were added, and the mixture was stirred for 2 h at 50 °C. It was then let to stand overnight so that the glycerol would settle in the bottom. The upper, liquid phase was transferred to a round bottom flask, and the methanol was removed under reduced pressure. The epoxy equivalent weight of epoxidized soybean oil methyl ester was determined to be 230 g/equiv. and 184 g/equiv. for the epoxidized linseed oil methyl ester.

### 3.3. Lignin Fractionation and Characterization

Solvent extraction was performed by dissolving 10 g of lignin in 150 mL of a solvent (methanol or acetone), stirring overnight, filtering through a Büchner funnel, followed by concentrating the filtrate and finally drying the lignin at 50 °C under vacuum for 16 h. The lignin hydroxyl content was determined by ^31^P NMR according to a literature procedure [27].

### 3.4. Preparation and Curing of Resins

An amount of lignin (LignoBoost Kraft or the solvent-extracted fractions) was dissolved in 2 mL of tetrahydrofuran and mixed with 1 g of epoxidized vegetable oil (linseed, soybean, or their methyl esters). Alternatively, ethanol can be used as a solvent. The mixture was stirred at 70 °C for 1 h in an open flask. Afterwards, an amount of Priamine 1074 was added, and the mixture was stirred for 1 min and poured in a Teflon mold. The Teflon mold was placed in a vacuum oven (850 mbar) at 50 °C for 1 h to allow the solvent to evaporate. Then, the Teflon mold was placed in an oven and cured at 120 °C for 4 h and at 150 °C for 16 h. The curing could also be done at 120 °C for 20 h in the presence of 1-methylimidazole as a catalyst without an influence on the outcome. When BCD Eucalyptus lignin was used, no solvent was required, and thus the required amount of lignin was mixed with 1 g of epoxidized vegetable oil and stirred at 70 °C for 30 min in an open flask. The rest of the protocol was as described above.

### 3.5. Chemical Analysis

Gel permeation chromatography was performed on a Styragel HR1 column eluting with tetrahydrofuran with RID detection. The hydroxyl content was quantitatively determined using ^31^P NMR spectroscopy, as described in the literature [27]. The epoxy equivalent weight of methyl esters of the epoxidized vegetable oils was determined by potentiometric titration following ASTM Standard D1652−04.

### 3.6. Thermal Analysis

Possible thermal phase transitions in all of the cured resin specimens were investigated using differential scanning calorimetry (DSC). The DSC measurements were conducted with TA Discovery DSC 250 by subjecting the cured epoxy resins to a heat–cool–heat cycle from −80 to 200 °C at a 20 °C/min rate under a nitrogen atmosphere. Normalized heat flows were plotted as a function of the temperature, and the plots were analyzed. The glass transition temperature (*T*_g_) was defined as the half value of the heat capacity change (ΔCp/2). Thermal gravimetric analyses (TGA) were performed to gain insight into the degradation temperature of the resins. TGA measurements were carried out in a nitrogen environment (flow rate 20 mL min^−1^) using a Netzsch STA 449c machine at a 10 °C min^−1^ heating rate from 25 to 800 °C.

### 3.7. Spectroscopic Analysis

Attenuated total reflectance-Fourier transform infrared (ATR-FTIR) analyses of the epoxy resins were performed using a Nicolet iS10 spectrometer (Thermo Fisher Scientific, Waltham, MA, USA) operating at laboratory atmospheric conditions. The measurements were performed in the attenuated reflectance (ATR) mode using a diamond crystal. The spectral range was 4000 to 500 cm^−1^. The scans were performed with a 4 cm^−1^ scan resolution and 32 scans per sample. Blank scans were subtracted from the sample scans prior to their analysis.

### 3.8. Tensile Test

The tensile tests of the cured epoxy resins were conducted according to ASTM D638, using an Instron machine model 2412 (Instron, England). Three replicates were measured for each sample, with the dimensions of ASTM D638 Type V. The tests were performed at 22 ± 2 °C and at 50–60% relative humidity. The crosshead speed was 2.5 mm/min. Young’s modulus, ultimate tensile strength, and percentage elongation at break were calculated from the stress-strain curves on the basis of the initial sample dimensions.

## 4. Conclusions

A series of fully biobased epoxy resins made from renewable resources were prepared, and their thermo-mechanical properties were analyzed. Lignin, rich in aromatic structures, gives stiffness and consistency to the epoxy resins, which are otherwise soft, flexible, and breakable due to long aliphatic chains of the fatty acid derivatives. Solvent extracted Kraft lignin was used due to its more defined profile. At a low lignin content, the effects were barely noticeable, but at a 12.5 weight% of lignin both the glass transition temperature and the Young’s modulus increased, and the rise was even more pronounced at a 22 weight%. It is thus not a linear but an exponential correlation. Higher amounts of lignin could not be incorporated if solvent-extracted Kraft lignin was used. Depolymerized and subsequently solvent-extracted lignin, liquid and with low Mw, could be used in higher amounts (36.4%), leading to harder epoxy resins. The fact that all the components of the resin are bulk chemicals opens the door to the production of fully biobased resins on an industrial level.

## 5. Patents

Ortiz, P.; Vendamme, R. Bio Based Epoxy Resin. PCT/EP2019/075067.

## Figures and Tables

**Table 1:** Characterization of lignin fractions.

Lignin Fraction	Yield (%)	Mw (g/mol) ^1^	PDI ^1^	*T*_g_ (°C) ^2^	Aliphatic OH (mmol/g) ^3^	Aromatic OH (mmol/g) ^3^	COOH (mmol/g) ^3^
KL	-	5200	3.5	132	2.0	4.3	0.4
KL_MeOH_	48	1610	2.0	89	1.7	4.5	0.5
KL_Acetone_	61	2540	2.2	114	1.5	4.6	0.5

^1^ Determined by GPC. ^2^ Determined by DSC. ^3^ Determined by ^31^P NMR.

**Table 2:** The effect of the diamine content on the thermomechanical properties of the epoxy resins.

Diamine Equiv. to ELO_me._	E_Young_ (MPa) ^1^	Σ_Break_(MPa) ^1^	Ε_Break_ (%) ^1^	*T*_g_ (°C) ^2^
0.75	22.1 ± 1.6	2.5 ± 0.1	47 ± 2	−2
1	13.8 ± 1.3	4.0 ± 0.7	114 ± 14	0
1.25	7.4 ± 0.4	2.2 ± 0.1	100 ± 33	−5
1.5	4.7 ± 1.1	2.2 ± 0.3	144 ± 15	−6

^1^ Calculated by tensile test. Data are the average of three test pieces ± SD. ^2^ Determined by DSC (S10 in Supplementary Materials).

**Table 3:** Influence of the lignin content on the mechanical properties of the cured epoxy resins.

KL_MeOH_ Content (% weight) ^1^	E_Young_ (MPa) ^2^	Σ_Break_(MPa) ^2^	Ε_Break_ (%) ^2^
0	0.26 ± 0.01	0.4 ± 0.1	197 ± 11
6	0.7 ± 0.1	0.6 ± 0.1	105 ± 15
12.5	2.2 ± 0.4	1.8 ± 0.4	129 ± 16
22.2	13.8 ± 1.3	4.0 ± 0.7	114 ± 14

^1^ Lignin content as the % weight of lignin in the resin. ^2^ Calculated by tensile test. Data are the average of three test pieces ± SD.

**Table 4:** Thermal decomposition data from the TGA curves.

Lignin Content (% weight) ^1^	2.5% Weight Loss (°C)	5% Weight Loss (°C)	Residual Mass at 800 °C (% weight)
0	249	302	1
12.5	257	292	14
22.2	276	315	40

^1^ Lignin content as the % weight of KL_MeOH_ in the resin.

**Table 5:** Effect of the lignin type on the thermomechanical properties of the epoxy resins.

Lignin Type	Lignin Content (% weight) ^1^	Mw (g/mol) ^2^	Dispersity ^2^	E_Young_ (MPa) ^3^	Σ_Break_ (MPa) ^3^	Ε_Break_ (%) ^3^	*T*_g_ (°C) ^4^
KL_MeOH_	22.2	1610	2.0	13.8 ± 1.3	4.0 ± 0.7	114 ± 14	0
KL_Acetone_	22.2	2540	2.2	20.9 ± 1.6	5.7 ± 0.4	121 ± 14	4
KL	22.2	5200	3.5	35.2 ± 9.0	4.1 ± 1.1	41 ± 6	0
West Fraser KL	22.2	4080	3.8	17.6 ± 0.8	6.8 ± 0.7	135 ± 13	0
BCD oil	22.2	330	1.4	31.1 ± 1.3	3.6 ± 0.2	21 ± 3	9
BCD oil	36.4	330	1.4	60.2 ± 9.6	6.5 ± 0.3	21 ± 8	15

^1^ Lignin content as the weight% of lignin in the resin. ^2^ Determined by GPC (Supplementary Materials S4–S6; S11–S12). ^3^ Calculated by tensile test. Data are the average of three test pieces ± SD. ^4^ Determined by DSC (Supplementary Materials S13).

## Supplementary Materials

The following are available online, ^31^P NMR characterization of lignin S1–S3; GPC characterization of lignins S4–S8; Proof of epoxy curing S9–S10; Plot of equiv. of diamine vs. Young’s Modulus of cured samples S11; DSC of cured samples S12–S13; TGA of cured samples S14.
