# Supplementary material for: Fully Biobased Epoxy Resins from Fatty Acids and Lignin
Source: Molecules. 2020 Mar 5;25(5):1158. doi: 10.3390/molecules25051158 (PMC7179237; doi:10.3390/molecules25051158)

# Fully biobased epoxy resins from fatty acids and lignin

*Pablo Ortiz* [1,2,*], *Richard Vendamme* [1,2] *and Walter Eevers* [1,3]

[1] Flemish Institute for Technological Research – VITO, Separation & Conversion Technology, Boeretang 200, 2400 Mol, Belgium. pablo.ortiz@vito.be; richard.vendamme@vito.be. walter.eevers@vito.be
[2] Biorizon, Auvergnedijk 2, 4612 PZ Bergen op Zoom, The Netherlands.
[3] Department of Chemistry, University of Antwerp, Groenenborgerlaan 171, B-2020 Antwerpen, Belgium.

# Characterization of lignins

## NMR

### S1 Kraft lignoboost

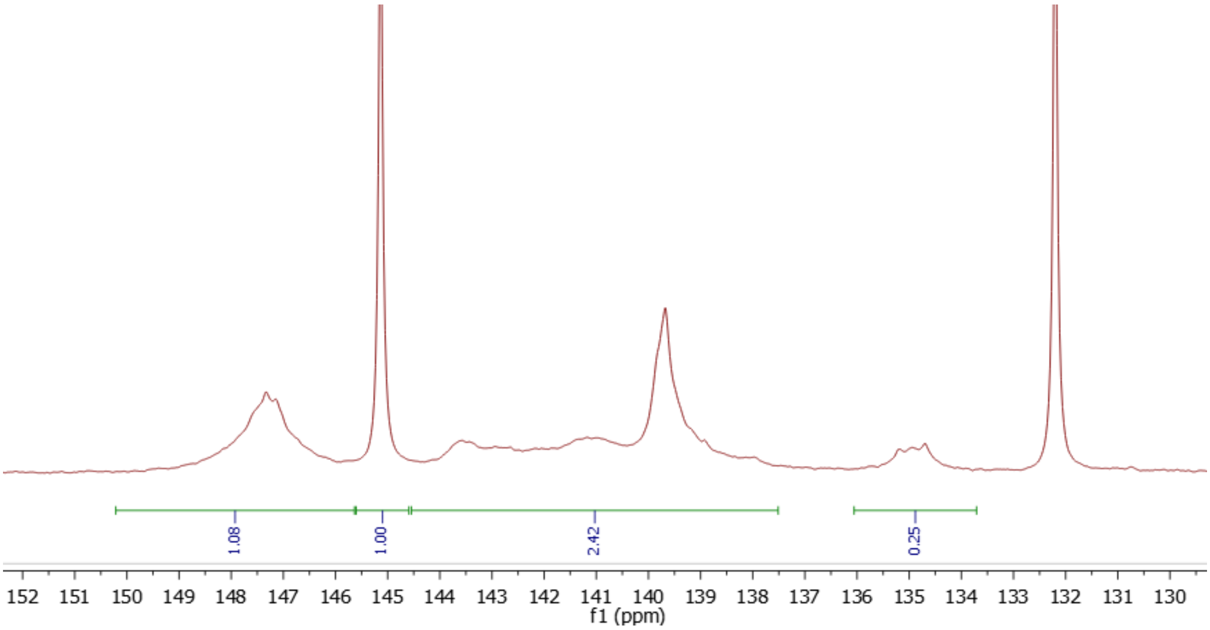


### S2 Acetone extracted Kraft

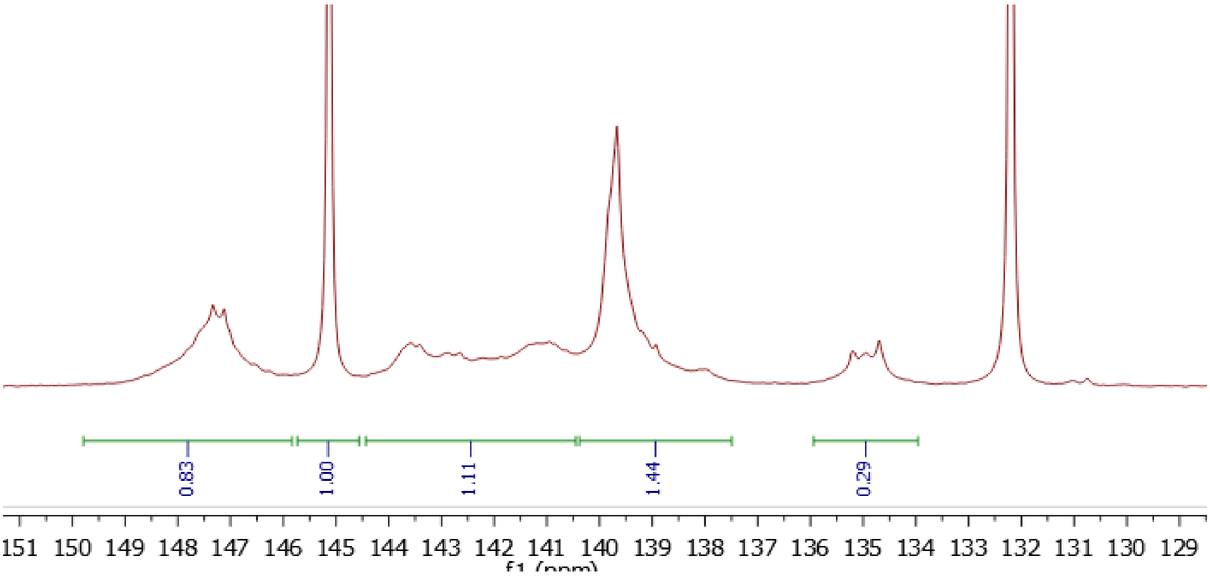

### S3 MeOH extracted Kraft

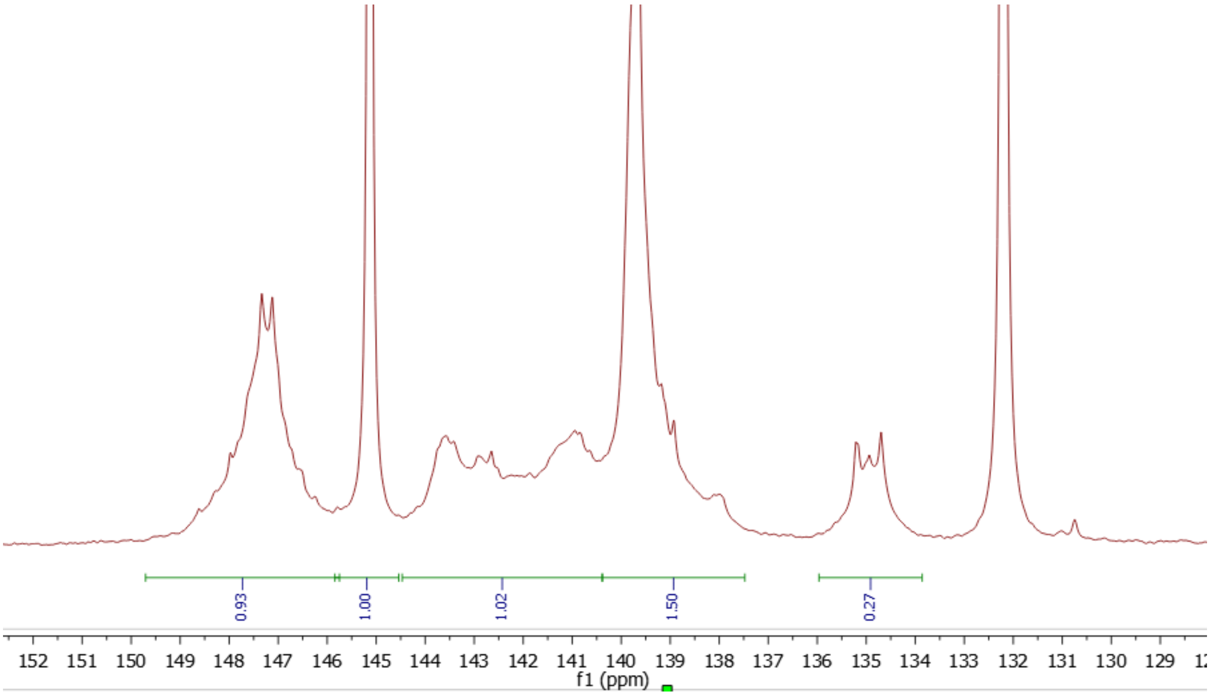


## GPC

### S4 Lignoboost KL

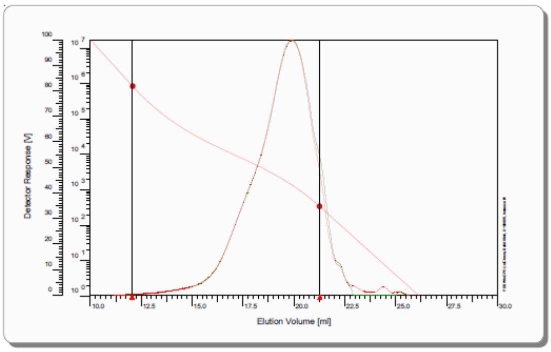


**Operator:** WinGPC01
**Sample name:** Vial 23: 190626-0164 KL - 1
**Project file:** C:\wingpc_8#1\GPC1\BioHart.LDX
**Calibration:** CalibTHF20190626HR3-4.cal (created 28.6.2019, 08:01)

**Inject date:** Thursday, 27.06.2019 00:06:00
**Concentration:** 2,000 g/l
**Baseline from:** 11,165 ml **to:** 25,678 ml **Integration from:** 12,054 ml **to:** 21,276 ml
**Int. Standard:** --- **at:** 50,000 ml

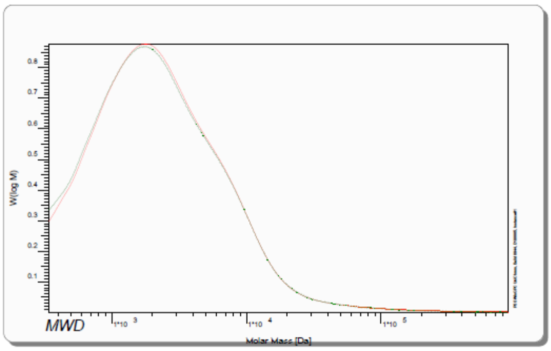


**Eluent:** THF
**Pump:** LC-20AT **Flowrate:** 0,80 ml/min
**Injector:** SIL-20AC **Inj. Vol.:** 100,00 µl
**Interface:** PSS/UDC 810 CH
**Column 1:** Styragel Guard Column
**Column 2:** Styragel HR1
**Column 3:** Styragel HR0.5
**Detector 1:** SPD-20A UV **Delay:** 0,000 ml
**Detector 2:** RID-10A **Delay:** 0,130 ml

| *Detector* | 1 | 2 |
|---|---|---|
| Name: | SPD-20A UV | RID-10A |
| Mn [Da]: | 1.450 | 1.420 |
| Mw [Da]: | 5.370 | 5.200 |
| Mz [Da]: | 99.600 | 89.000 |
| Mv [Da]: | | |
| D: | 3,713 | 3,662 |

## S5 Lignoboost KL – MeOH extracted

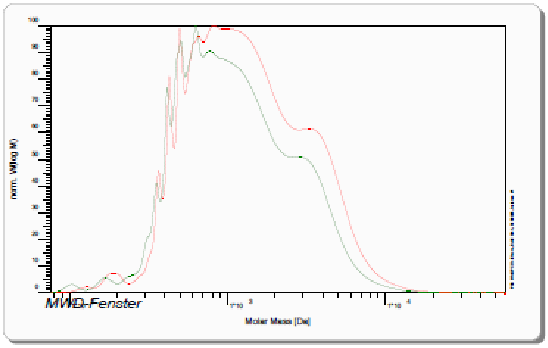


| | | | |
|---|---|---|---|
| **Eluent:** | THF | | |
| **Pump:** | LC-20AT | **Flowrate:** | 0,80 ml/min |
| **Injector:** | SIL-20AC | **Inj. Vol.:** | 25,00 µl |
| **Interface:** | PSS/UDC 810 CH | | |
| **Column 1:** | Ultrahydrogel Guard Column | | |
| **Column 2:** | Ultrahydrogel 500 | | |
| **Column 3:** | Ultrahydrogel 120 | | |
| **Detector 1:** | SPD-20A UV | **Delay:** | 0,000 ml |
| **Detector 2:** | RID-10A | **Delay:** | 0,000 ml |

| *Detector* | *1* | *2* |
|---|---|---|
| **Name:** | **SPD-20A UV** | **RID-10A** |
| **Mn [Da]:** | 939 | 820 |
| **Mw [Da]:** | 1.880 | 1.610 |
| **Mz [Da]:** | 3.870 | 3.430 |
| **Mv [Da]:** | | |
| **D:** | 2,004 | 1,959 |

## S6 Lignoboost KL – Acetone extracted

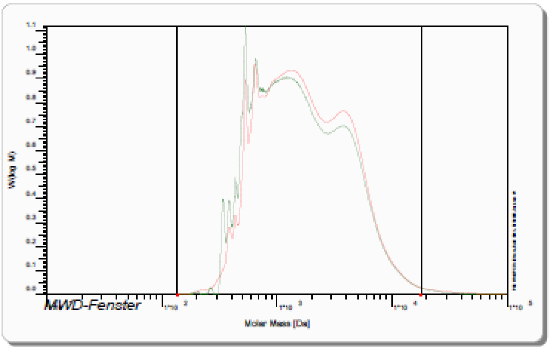


| | | | |
|---|---|---|---|
| **Eluent:** | THF | | |
| **Pump:** | LC-20AT | **Flowrate:** | 0,80 ml/min |
| **Injector:** | SIL-20AC | **Inj. Vol.:** | 100,00 µl |
| **Interface:** | PSS/UDC 810 CH | | |
| **Column 1:** | Styragel Guard Column | | |
| **Column 2:** | Styragel HR1 | | |
| **Column 3:** | Styragel HR0.5 | | |
| **Detector 1:** | SPD-20A UV | **Delay:** | 0,000 ml |
| **Detector 2:** | RID-10A | **Delay:** | 0,110 ml |

| *Detector* | *1* | *2* |
|---|---|---|
| **Name:** | **SPD-20A UV** | **RID-10A** |
| **Mn [Da]:** | 1.240 | 1.140 |
| **Mw [Da]:** | 2.740 | 2.540 |
| **Mz [Da]:** | 8.420 | 7.120 |
| **Mv [Da]:** | | |
| **D:** | 2,206 | 2,216 |

## S11 West Fraser KL

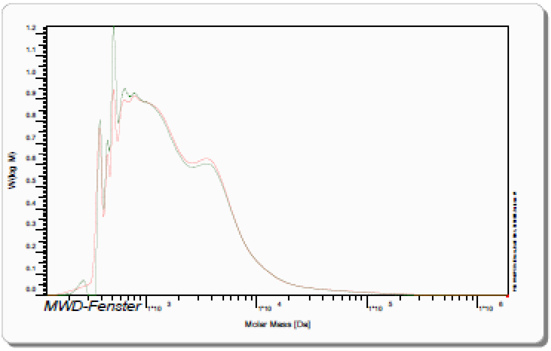


| | | | |
|---|---|---|---|
| **Eluent:** | THF | | |
| **Pump:** | LC-20AT | **Flowrate:** | 0,80 ml/min |
| **Injector:** | SIL-20AC | **Inj. Vol.:** | 100,00 µl |
| **Interface:** | PSS/UDC 810 CH | | |
| **Column 1:** | Styragel Guard Column | | |
| **Column 2:** | Styragel HR1 | | |
| **Column 3:** | Styragel HR0.5 | | |
| **Detector 1:** | SPD-20A UV | **Delay:** | 0,000 ml |
| **Detector 2:** | RID-10A | **Delay:** | 0,110 ml |

| *Detector* | *1* | *2* |
|---|---|---|
| **Name:** | **SPD-20A UV** | **RID-10A** |
| **Mn [Da]:** | 1.090 | 1.090 |
| **Mw [Da]:** | 4.570 | 4.080 |
| **Mz [Da]:** | 94.800 | 69.800 |
| **Mv [Da]:** | | |
| **D:** | 4,170 | 3,751 |

## S12 BCD, solvent extracted eucalyptus lignin

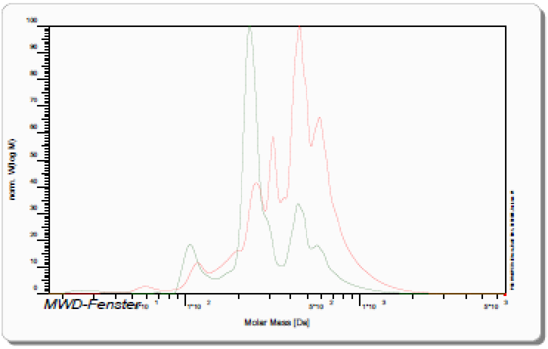


| | | | |
|---|---|---|---|
| **Eluent:** | THF | | |
| **Pump:** | LC-20AT | **Flowrate:** | 0,80 ml/min |
| **Injector:** | SIL-20AC | **Inj. Vol.:** | 40,00 µl |
| **Interface:** | PSS/UDC 810 CH | | |
| **Column 1:** | Styragel Guard Column | | |
| **Column 2:** | Styragel HR1 | | |
| **Column 3:** | Styragel HR0.5 | | |
| **Detector 1:** | SPD-20A UV | **Delay:** | 0,000 ml |
| **Detector 2:** | RID-10A | **Delay:** | 0,000 ml |

| *Detector* | *1* | *2* |
|---|---|---|
| **Name:** | **SPD-20A UV** | **RID-10A** |
| **Mn [Da]:** | 321 | 220 |
| **Mw [Da]:** | 466 | 327 |
| **Mz [Da]:** | 638 | 483 |
| **Mv [Da]:** | | |
| **D:** | 1,451 | 1,485 |

# Curing mechanism

## S7 Proof of reaction of lignin with the epoxy groups of $ELO_{me}$ by DSC

0.5 grams of BCD Eucaliptus lignin oil was mixed with 0.5 grams of $ELO_{me}$ and stirred for 1 minute at room temperature. Then an aliquot of the mixture was placed in DSC pan and heated at 10°C/min to 120°C. It was maintained at that temperature for 60 min. Reference samples of the $ELO_{me}$ as well as Eucaliptus BCD lignin oil were run at the same conditions Eucaliptus BCD lignin instead of Kraft lignin because it could be mixed with the $ELO_{me}$ without the need of solvents.

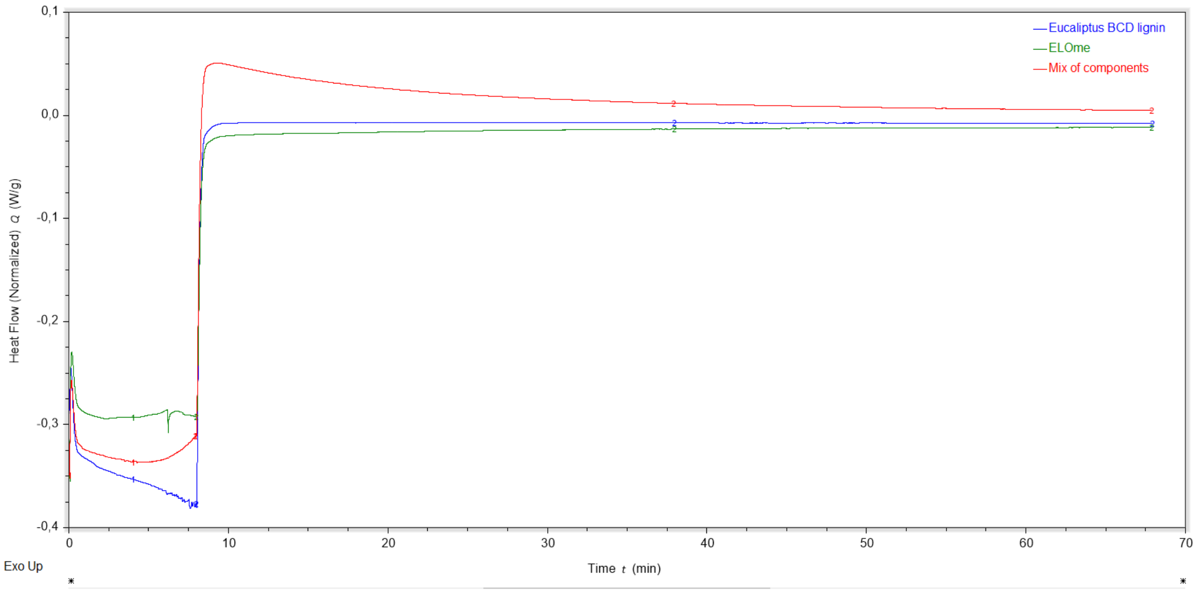

## S8 Proof of lignin incorporation in the resin by analyzing the leaching of a fully cured epoxy sample by $^{1}H$ NMR

150 mg of the fully cured epoxy resin with 22% of $KL_{MeOH}$ was immersed in a vial containing 15 ml of THF and was kept there for 48h. The sample was removed, and the THF evaporated. The residue was analyzed by $^{1}H$ NMR using $CDCl_3$ as solvent.

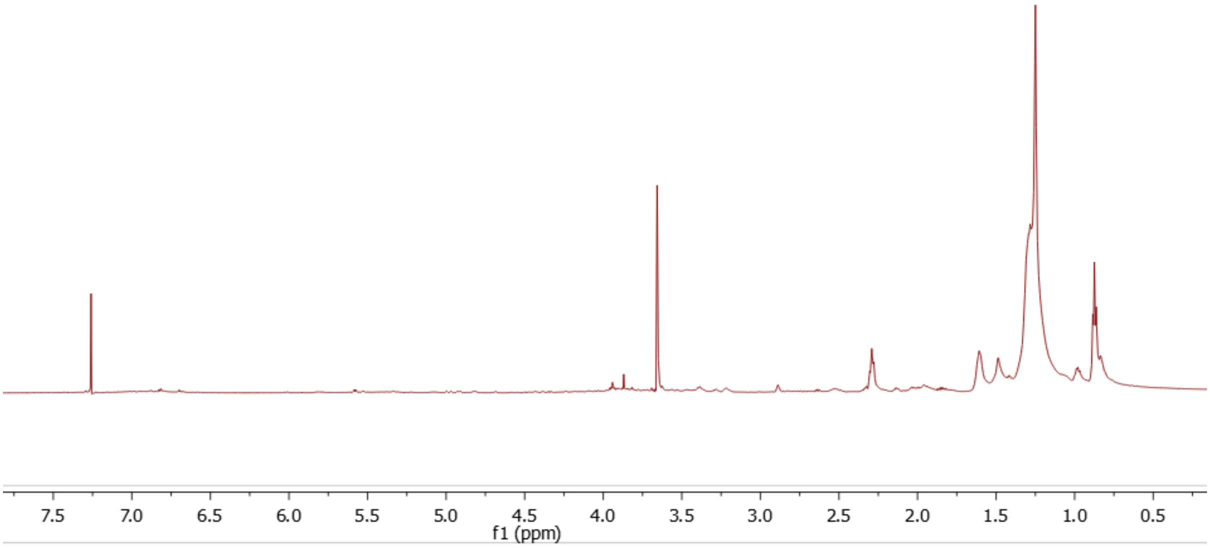

# Characterization of cured samples

**S9 Plot of equivalents of diamine vs Young's modulus**

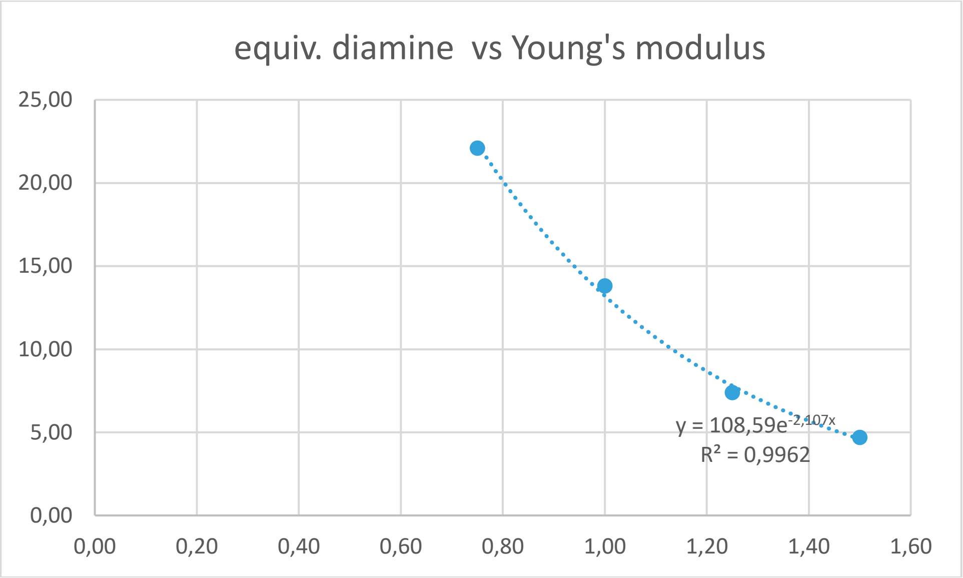


## DSC-2nd heating curves

**S10 Variable amounts of Dimer diamine**

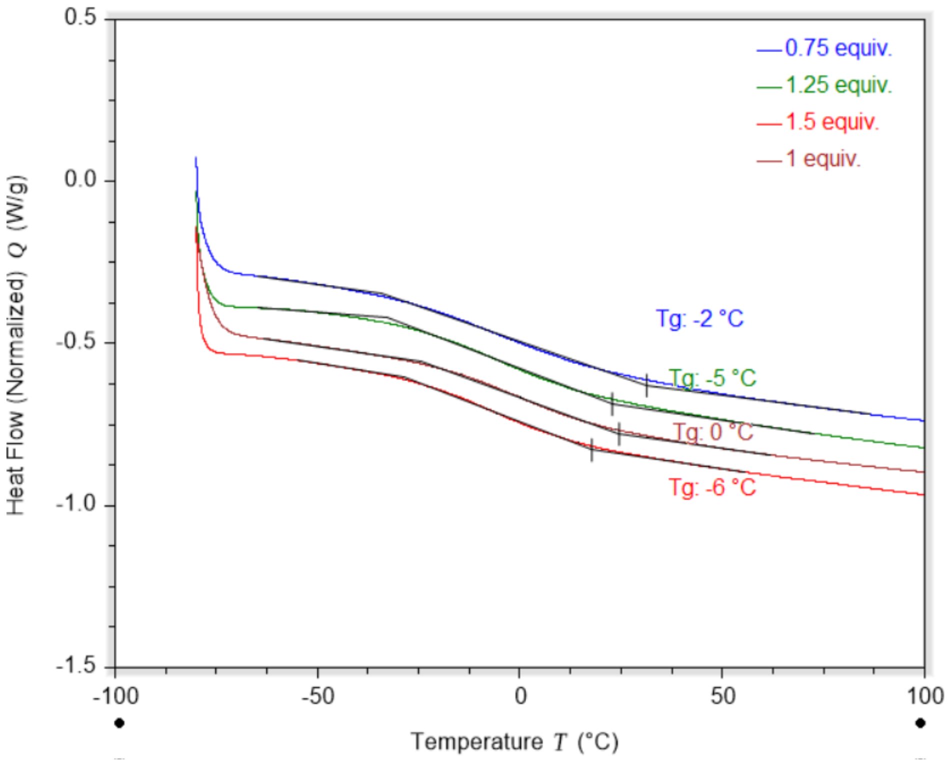

**S13 Different types of lignins**

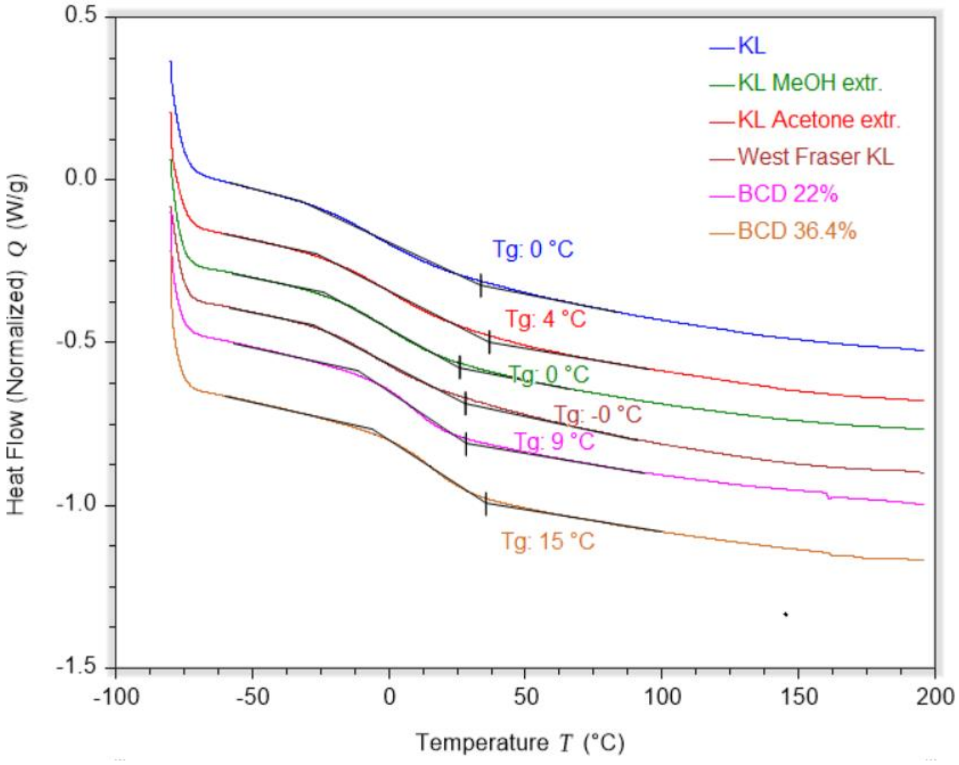


# S14 TGA

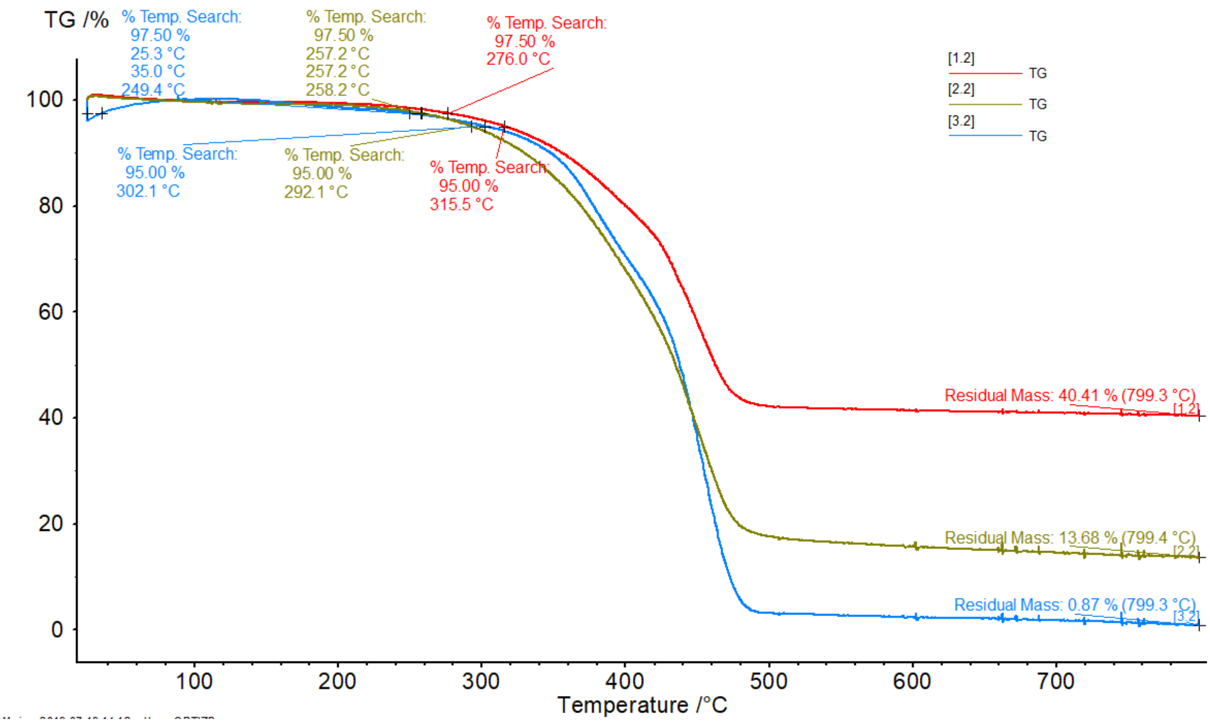

Supplement: Supplementary file 1 [file molecules-25-01158-s001.zip › molecules-732466-supplementary.pdf]
